# From documents to datasets: A MediaWiki-based method of annotating and extracting species observations in century-old field notebooks

**DOI:** 10.3897/zookeys.209.3247

**Published:** 2012-07-20

**Authors:** Andrea Thomer, Gaurav Vaidya, Robert Guralnick, David Bloom, Laura Russell

**Affiliations:** 1 University of Illinois, Urbana-Champaign, Graduate School of Library and Information Science, 501 E. Daniel Street, Champaign, Illinois, 61820, USA; 2 University of Colorado, Boulder; University of Colorado Museum of Natural History, Henderson Building, Boulder, Colorado, 80309, USA; 3 University of California, Berkeley, Museum of Vertebrate Zoology, 3101 Valley Life Sciences Building, Berkeley, California, 94705, USA; 4 University of Kansas, KU Biodiversity Institute, 1345 Jayhawk Blvd., Room 606, Lawrence, Kansas, 66045, USA

**Keywords:** Field notes, notebooks, crowd sourcing, digitization, biodiversity, transcription, text-mining, Darwin Core, Junius Henderson, annotation, taxonomic referencing, natural history, Wikisource, Colorado, species occurrence records

## Abstract

Part diary, part scientific record, biological field notebooks often contain details necessary to understanding the location and environmental conditions existent during collecting events. Despite their clear value for (and recent use in) global change studies, the text-mining outputs from field notebooks have been idiosyncratic to specific research projects, and impossible to discover or re-use. Best practices and workflows for digitization, transcription, extraction, and integration with other sources are nascent or non-existent. In this paper, we demonstrate a workflow to generate structured outputs while also maintaining links to the original texts. The first step in this workflow was to place already digitized and transcribed field notebooks from the University of Colorado Museum of Natural History founder, Junius Henderson, on Wikisource, an open text transcription platform. Next, we created Wikisource templates to document places, dates, and taxa to facilitate annotation and wiki-linking. We then requested help from the public, through social media tools, to take advantage of volunteer efforts and energy. After three notebooks were fully annotated, content was converted into XML and annotations were extracted and cross-walked into Darwin Core compliant record sets. Finally, these recordsets were vetted, to provide valid taxon names, via a process we call “taxonomic referencing.” The result is identification and mobilization of 1,068 observations from three of Henderson’s thirteen notebooks and a publishable Darwin Core record set for use in other analyses. Although challenges remain, this work demonstrates a feasible approach to unlock observations from field notebooks that enhances their discovery and interoperability without losing the narrative context from which those observations are drawn.

“Compose your notes as if you were writing a letter to someone a century in the future.”

Perrine and Patton (2011)

## Introduction

Our species has analyzed and documented the natural world for millennia, in media as diverse as Paleolithic cave paintings, handwritten field notes, and structured databases of sequences sampled from the environment. While structured data facilitate long-term ecological monitoring, the “first-person precision” (Grinnell 1912) of an idiosyncratic, unatomizable narrative about nature — be it a drawing on a cave wall or a handwritten page in a field journal — gives these data context that does not readily fit into a spreadsheet, and which may form the nucleus of an important new insight or discovery. Field notes in particular sit at the crossroads of these qualitative and quantitative methods; in them, structured and unstructured data are necessarily intertwined (Kramer 2011).

The observations contained in field notebooks take on particular importance given the current biodiversity crisis (Jenkins 2003, Heywood and Watson 1995, Loreau et al. 2006, Wake and Vredenburg 2008) — a crisis which threatens the fabric of ecosystems on which our own species depends (e.g. Millennium Ecosystem Assessment 2005, Worm et al. 2006). Legacy occurrence records extracted from field notebooks provide essential baselines of past community biotic state for resurvey efforts such as the Grinnell Resurvey Project (Moritz et al. 2008, Tingley et al. 2009) and the Alexander Grasshopper Project (Nufio et al. 2010).

The growing use of such records for global change biology creates new challenges and opportunities for their digitization, transcription, representation, and integration with other sources of historical data. All these challenges ultimately depend on pulling structured data from unstructured text, while somehow maintaining a link to the original texts. Solving these challenges is key to realizing their value in research and policy-making.

Here we present a case study that makes occurrence records in field notebooks available by utilizing something of a rarity in this arena: a fully scanned and transcribed set of field notebooks, penned by University of Colorado Museum of Natural History founder Junius Henderson (http://en.wikisource.org/wiki/Field_Notes_of_Junius_Henderson). We provide a pragmatic approach for utilizing free, relatively easy-to-use technologies to annotate these notes, and discuss some of the remaining gaps in our toolkits and cyberinfrastructure. We also present a workflow for extracting occurrence records from field notebooks that requires minimal resources (beyond the authors’ time), fosters community involvement, and abstracts the necessary information while maintaining links to its original text, thereby preserving the context that only “first-person precision” can provide. The primary challenges we address are how to: 1) publish these field notes in a way that supports annotation of species occurrence records; 2) extract these records efficiently; 3) convert these records to the most interoperable format; and, 4) store these records and maintain their link to the original field notes.

### Background

Remsen et al. (2012) identified conversion of unstructured text into structured data as a key challenge in biodiversity informatics, and showed a working methodology for creating a Darwin Core archive from a conventional floristic checklist. We follow the path laid by those authors, but focus on mining observations from field notebooks. Field notebooks are often “hidden” in archives of institutions, and unlike formally published sources, typically lack a centralized access point (Sheffield et al. 2011), a standardized mark-up language, and any sort of reliable or scalable method of mining content from the notes. Sheffield and Nakasone (2011) from the Smithsonian’s *Field Book Project* present an excellent high-level view of how existing metadata standards could be used to semantically link collections and field notes. This collections-level schema, however, does not address the need to annotate and extract data from documents. Furthermore, though work has been done linking digital collections to Wikipedia articles (*e.g*., Lally and Dunford 2007), and though the National Archives have recently partnered with Wikisource to upload their materials for transcription (http://transcribe.archives.gov/), neither of these projects have attempted to annotate or extract data from the materials.

In light of this lack of prior work, and given the observational nature of the notes, we decided that these observations would be best published as Darwin Core records. Though there are other standards used in the digital humanities to mark up scholarly texts (e.g. the Text Encoding Initiative’s standard, http://www.tei-c.org/), none of these are tailored for the encoding of biodiversity data. Darwin Core, on the other hand, is a commonly used metadata schema for describing and exchanging a range of biodiversity data, from museum specimen records to field observations (Wieczorek et al. 2012). In particular, the Global Biodiversity Information Facility (GBIF) uses it for storage, transfer and presentation of biodiversity data.

### The study corpus: Junius Henderson’s field notes

**Figure 1.**
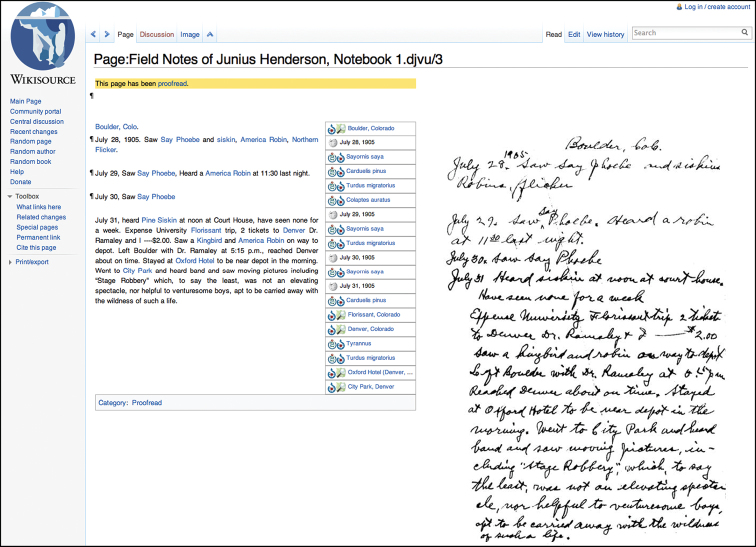
Web browser view of a scanned page of Henderson’s journal displayed side-by-side with transcriptions and annotations using the MediaWiki *Proofread Page* extension.

Junius Henderson was appointed the first curator of the University of Colorado Museum of Natural History (CU Museum) in 1902. He kept handwritten field notebooks describing his expeditions across the Southern Rocky Mountains and elsewhere over a 26-year period. Henderson completed 13 notebooks and 1,672 pages of entries, augmented by other materials such as photographs and a locality ledger. Henderson’s notes are arranged as entries (Figure 1), which usually contain some kind of header denoting date and place. All entries are separated by a blank space, so even if header text is not strictly standardized, the beginning and end of each entry is quite clear. Although Henderson did keep a locality ledger, he did not directly or systematically reference specimens to field note entries. Thus, if there are direct links between collected specimens and field notes, they have yet to be discovered.

Henderson’s notebooks are a chronicle of the American West in transition and paint a vivid picture of a changing landscape as cities expand, wild places retreat, and horse-and-buggies give way to cars. His journal entries describe everything from mollusks in freshwater and marine systems, to the geology of the Rocky Mountains, to the more mundane aspects of fieldwork (e.g., “Train again so late as to afford ample opportunity for philosophic meditation upon the motives which inspire railroad people to advertise time which they do not expect to make except under rare circumstances,”) (Henderson 1907).

From February 2000–02, former CU Museum Director and Curator Peter Robinson transcribed all thirteen volumes of Henderson’s notes into Word documents — a herculean task given Henderson’s handwriting. In 2006, the National Snow and Ice Data Center (NSIDC) scanned Henderson’s thirteen notebooks for a large glaciology project. Through a lengthy series of events, documented more fully in a series of blog posts (http://bit.ly/jhfnblog), the scans and transcriptions, separated from each other for several years, were reunited once we began work on this project.

The existence of both scanned images and typed transcriptions made Henderson’s notes an excellent test case for annotation and automated occurrence extraction; transcriptions could be tagged and annotated via a markup schema, and checked against scanned images of the original pages to ensure accuracy. As of this writing, only the first three notebooks have been annotated.

## Methods

We documented this project using a blog as an open notebook and a means to communicate our goals, ideas, and progress. Those goals were: (a) to make Henderson’s notes easily discoverable, publicly accessible, freely reusable and sustainably preserved and, and (b) to extract taxonomic occurrences from these notes.

### A platform for field notebook access and annotation: Wikisource

We quickly realized we needed a way to support the annotation of species occurrences on an open platform so that anyone interested could help with the task. We decided on the Wikipedia-related project Wikisource (http://wikisource.org) for the following reasons:

#### Ease of use.

The process of uploading scanned pages is simple. PDFs are uploaded to the Wikimedia Commons and pulled into Wikisource. Once in Wikisource, hyperlinked index pages can be created and transcribed text can be matched with the scanned image of each field book page (Figure 1). The wiki markup language is similarly easy to learn and use. The language is the same as that used in Wikipedia, which means skills developed in Wikipedia can be brought to Wikisource easily.

#### Completely open access.

Everything on Wikisource can be edited by anyone, giving us a way to crowdsource annotation to citizen scientists and archivists. All Wikisource pages have a built-in means of tracking edits that ensure that all changes made to the transcriptions are documented and reversible.

#### An existing community of developers.

Wikisource uses the same software as Wikipedia (a PHP application named “MediaWiki”), which is under active development by a core team of developers. Sharing the same software and licensing terms means that content can be shared between the two projects freely. Additionally, pages designed to be incorporated into other pages (known as *templates* in Wikispeak; see http://en.wikipedia.org/wiki/Template:Cleanup for an example) can be moved from one project to another easily, speeding development. The Wikipedia community also carries out software development for Wikisource-specific features; our project relied on the *Proofread Page* extension to provide side-by-side views of transcriptions and their corresponding scanned images (Figure 1).

#### An existing community of users, transcribers, and proofreaders.

There is an active Wikisource community improving Wikisource’s content and to transcribing newly uploaded texts (see http://en.wikisource.org/wiki/Wikisource:Community_collaboration). We hoped to draw some of these community members into our project.

### Uploading content

The ideal upload to Wikisource is a Portable Document Format (PDF) or DjVu multipage image file containing the entire scanned document along with its OCRed text (sometimes referred to as a “searchable PDF”). Such files retain their text in Wikisource, making transcription easy. In our case, we uploaded handwritten scans as-is and inserted the transcriptions manually. PDF or DjVu files are uploaded to the Wikimedia Commons using the Upload Wizard (http://bit.ly/wcupload) and reused in Wikisource. One important note: both the Wikimedia Commons and Wikisource only allow the upload of materials in the public domain or published under liberal open source licenses (such as the Creative Commons Attribution or Creative Commons Attribution-ShareAlike licenses). Materials that have only been made available for non-commercial use may not be uploaded to the Wikimedia Commons. This means that data from the Biodiversity Heritage Library, which uses a Creative Commons Non-Commercial Share-Alike license, could not be uploaded to Wikisource. For a thorough discussion of the effect of these licenses on biodiversity science, see Hagedorn et al. (2011).

**Figure 2.**
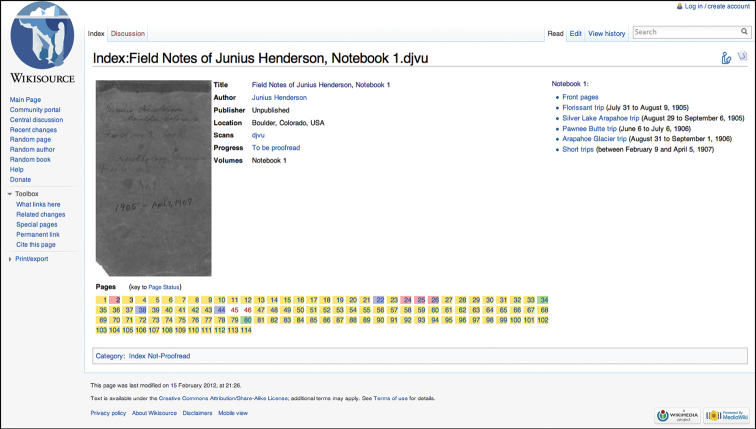
Index page for Notebook #1. Each Index page corresponds to a multipage file. The Index page displays volume metadata and links to sections of the notebook, while also providing links out to each notebook page and color-coding to determine which pages have been already transcribed and proofed.

While uploading images to the Commons is simple, reusing them in Wikisource can be tricky (a guide to this process — updated by us — is available on Wikisource: http://bit.ly/wsindexhelp). After setting up the Index page (Figure 2) and copying the transcriptions into Wikisource manually, we were ready to begin annotation.

### Creating annotation templates

In Wikisource, annotations are best made through the use of templates. Templates are a feature of the MediaWiki software that allows one wiki page to be inserted into another. While usually used to embed common design elements across Wikipedia (such as the *Unbalanced* template, used to warn readers that an article might be unbalanced: http://en.wikipedia.org/wiki/Template:Unbalanced), they can also provide complex functionality, such as creating a standardized citation format (see http://en.wikipedia.org/wiki/Template:Cite_journal) or calculating ages from birthdates. We developed our own templates to not only tag the elements of an occurrence record but also create links to other web resources.

### The elements of an occurrence record

A species occurrence record should contain the following basic elements in order to be fit-for-use in biodiversity science: 1) the species’ name, and 2) the place and 3) time in which it was observed. Also important, but slightly less crucial, is additional information describing the observation event: the name of the person making the observation, any equipment used, the sampling method, and so on.

Thus, because our goal was the extraction of occurrence records, we created annotation templates for *taxa*, *locations*, and *dates*. A triplet of all three annotations would, in theory, be attributable to an observation event and could be pulled from the annotated text as an occurrence record. The templates link these elements to Wikipedia pages, and provide a means to show annotations separately from the text itself.

**Figure 3.**
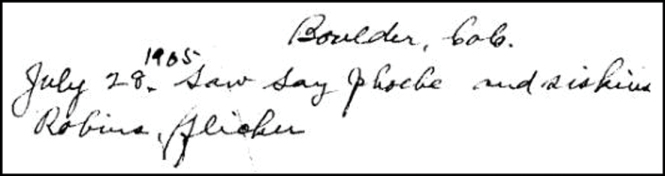
Henderson’s first sentence. “Boulder, Colo. July 28, 1905. Saw Say [sic] Phoebe and siskins, [American] Robins, [Northern] Flicker.”

The first sentence of Henderson’s first field book contains a simple example of the type of text we hoped to annotate with Wiki markup (Figure 3):

This single sentence contains six annotatable terms: a *location* (Boulder, Colo), a *date* (July 28, 1905), and four *taxa* (Say['s] Phoebe, Pine Siskin, American Robin, Northern Flicker). Each template attempts to link the annotated element to associated pages in the Wikimedia Commons and Wikipedia. Thus, templates include the verbatim text from Henderson and an interpretation of that element’s formal name (as determined by the annotator) that resolves to other Wiki-resources. The general syntax of these templates is:

{{*element*|f*ormal name of this element*|*element as written by Henderson*}}

For example, the first taxon annotation in the text reads:

{{*taxon*|*Sayornis saya*|*Say Phoebe*}}

While the process of creating these annotations is relatively simple, we soon discovered that each requires substantial decision making on the part of the annotator, leaving ample room for variation.

In the case of the “Siskin” above, annotators could make several interpretations.An experienced birder may reason that based on Henderson’s location at that time, he is referring to a Pine Siskin (*Carduelis pinus* and create the following annotation:

{{taxon|*Carduelis pinus*|siskins}}

But it’s just as likely that a less experienced annotator would create the following less specific, though technically correct, annotation:

{{taxon|Siskin|siskins}}

This latter annotation links to a Wikipedia disambiguation page listing 18 different bird species, a kind of British aircraft, and a Canadian junior ice hockey team (http://en.wikipedia.org/wiki/Siskin).

**Figure 4.**
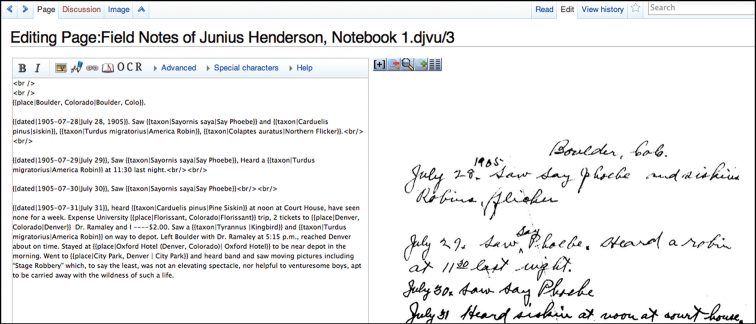
Editing a notebook page on Wikisource. This screenshot shows side-by-side transcription and wiki markup syntax.

We allowed our annotators complete flexibility in interpreting vernacular names as they saw fit while editing notebook pages (Figure 4); this meant that we had to review and resolve taxonomic annotations to a best valid taxon name, just as a lab supervisor would need to check a volunteer's work in a museum. In future work, we will take steps to prescribe best practices based on what we learned in this pilot project.

The full process of determining a valid scientific name from Henderson’s verbatim description is *taxonomic referencing*, analogous to georeferencing for localities. As with georeferencing, there is uncertainty in the process of linking legacy observations to current valid names; the level of uncertainty depends on who did the referencing and when. We discuss our approach to taxonomic referencing below.

### Data extraction: Seeking efficiency and accuracy

The annotated text from Henderson’s first three notebooks was downloaded using the MediaWiki API (http://bit.ly/mediawikiapi). Individual annotations were then identified using regular expressions. We have described this process in detail in supplementary file 1: “Methods Supplement_Henderson.pdf.” The Perl module and scripts used for this process are available at https://github.com/gaurav/henderson.

**Figure 5.**
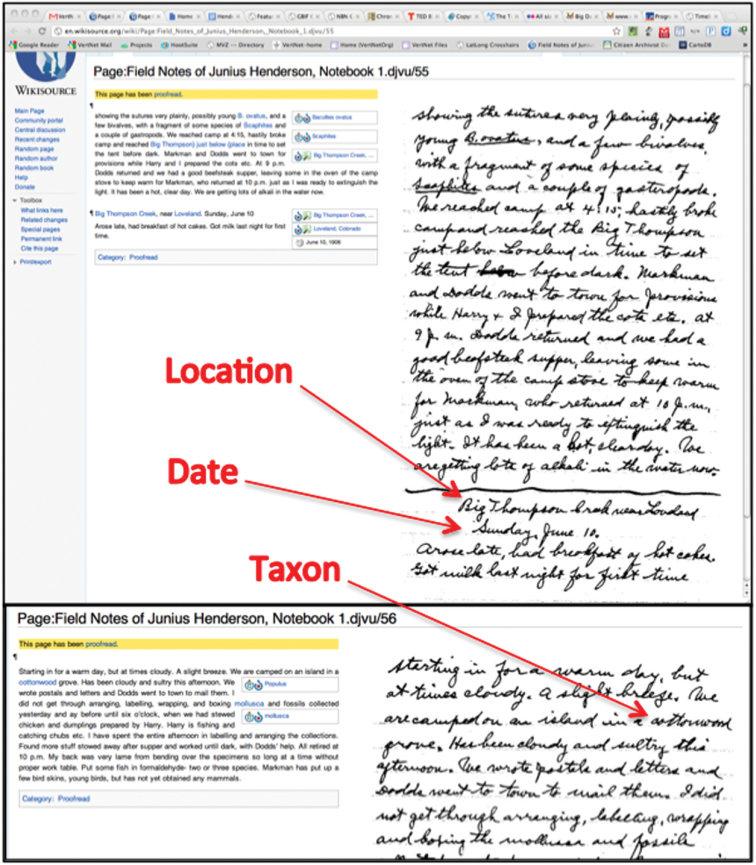
An example of how a location (Big Thompson Creek near Loveland), a date (Sunday, June 10, 1906), and a taxon (Cottonwood, genus Populus) are grouped from across multiple pages.

In summary, the steps were to:

1) Retrieve the number of pages in the file; 2) Extract the wiki markup from each individual page; 3) Write the wiki markup to a single XML file, which was divided into individual pages; 4) Concatenate this page-by-page file into one single text file to account for entries split across pages (Figure 5); 5) Divide the file into entries rather than pages; and 6) walk through the file, keeping track of the last location and date annotation encountered. Each taxon in an entry, coupled with the entry date and the preceding location, was tagged as an occurrence. Each triplet of elements that made up the occurrence was written to a CSV file, along with some text from the entry itself, the page number in the notebook, and a permanent link to the version of the Wikisource page containing the entry at the time the XML file was downloaded.

### Converting records into interoperable formats

After pulling occurrences into a CSV, we cross-walked this data into several fields selected from the Darwin Core Standard and added whatever supplementary information we could (e.g. by extrapolating higher taxonomy; see Appendix 1). Content in most fields depended on the four variables extracted from our dataset (taxon, date, location, page number), though some content was fixed (e.g., recordedBy always read “Junius Henderson”), and other content required manual determination or validation before being entered.

### Proofing the Darwin Core record set

The process of extracting taxon-location-date triplets is imperfect and requires vetting by proofreaders to ensure accuracy of the automated process, which does not consider contextual data. For example, our automated extraction scripts would incorrectly assume the following passage refers to a presence, not an absence: “Am perplexed by the entire absence of robins on this trip” (http://bit.ly/jhfn1-43). In future work, we plan to alter our templates to give annotators the ability to record whether an observation marks a presence or absence of a taxon.

As mentioned above, taxonomic names need special vetting, too. Henderson freely mixed vernacular and scientific names in his notes, and annotators consequently did as well. We performed taxonomic referencing using Google Refine, Encyclopedia of Life (EOL), and Integrated Taxonomic Information System (ITIS) name resolvers, following instructions from an *iPhylo* blog post by Rod Page (http://iphylo.blogspot.com/2012/02/using-google-refine-and-taxonomic.html). First, we loaded our CSV files from each field notebook into Google Refine. We then reconciled names assigned by annotators against the ITIS Freebase namespace (integrated within Google Refine) and the EOL service (developed by Page), and accepted the best judgments (as determined by probability scores). Those *best names* from each service were placed into two separate columns for further expert validation. The rows that produced consistent results from both EOL and ITIS name services were considered correct after a quick check for accuracy. One of the authors (Vaidya) checked each record in which EOL and ITIS suggested different best names and either chose the EOL name, the ITIS name, both, or neither. In many cases, one service provided a clear best fit at the right taxonomic depth compared to the other. In cases where both provided poor results, we did not choose a name. On those records where ITIS was found to be the best fit, we used the ITIS Taxonomic Serial Number to populate the vernacularName and the higher taxonomy fields. We also recorded the taxonomic resolution service used (EOL, ITIS, or EOL & ITIS) in the identificationRemarks field of the Darwin Core file we produced.

We also checked for annotation errors directly on Wikisource. One of the authors (Guralnick) went through each page of Notebook 1 on Wikisource to check for any obvious problems, such as poor formatting, mislabeling, or missed annotations (e.g., dates, locations, or taxa that could have been annotated but were not). He also checked all three notebooks for annotations that noted absences or that otherwise were not obviously observations.

### Data archiving and maintaining links to the original notes

All generated Darwin Core occurrence records include a URL to the page in Wikisource from which they are drawn in the Source field, i.e., they will take you to the version of the page that was live at the time at which the original XML file was created, not the latest version of the file. Additionally, each record is assigned an automatically generated catalog number as the record is extracted from the notebook.

## Data resources

The data presented in this paper are available for download in a Darwin Core Archive via VertNet, http://ipt.vertnet.org:8080/ipt/resource.do?r=hendersonnotebooks1-3. The archive includes taxon occurrences extracted from the field notes of Junius Henderson as he traveled through Colorado and the western United States.

## Results

After advertising our project via the blog, Twitter, and emails to relevant listservs, a total of three notebooks were transcribed and annotated, largely by volunteers (Table 1): 352 pages of notes and 222 entries in all. As of March 27, 2012, 10 registered Wikisource users and 11 anonymous users helped annotate these notebooks. All three notebooks were annotated within four to six weeks each. Again, only three of Henderson’s thirteen notebooks were uploaded for the purposes of this pilot project; we hope to upload and annotate the remaining notebooks soon.

A total of 1,087 taxon annotations were created across all three books, with each entry having between zero and 33 taxon annotations. Taxonomic resolution led to 560 records that were identified as valid by both EOL and ITIS taxonomic name resolvers. Expert validation led to 195 records as judged to be matched better by EOL than ITIS, and 83 records wherein the ITIS match was preferable to EOL’s. A total of 238 records could not be validated by either EOL or ITIS.

In Notebook 1, only two of 634 annotations were poorly formatted, caused by missing brackets. Only one date was transcribed incorrectly: “Apl 5/07” was annotated incorrectly as “April 7, 1907” (http://bit.ly/enws3614593). Also in Notebook 1, ten places and taxa could have been annotated but were not, and in all cases these were very broad taxonomic groups (e.g., Crustacea). A total of eleven taxon annotations across all three notebooks were manually identified as not denoting presence, and removed from the final dataset. Overall, the error rates and false positives were very low. After eliminating records of absence and some incorrect annotations, 1,068 valid observations remained; these were exported to a final Darwin Core Archive is included in the supplemental materials of this paper (see supplemental file 2: “dwca-hendersonnotebooks1-3.zip”).

**Table 1.** Summary information on each notebook.

	Notebook 1	Notebook 2	Notebook 3
URL	http://bit.ly/jhfn1-indexpg	http://bit.ly/jhfn2-indexpg	http://bit.ly/jhfn3-indexpg
Number of annotations	632	703	1007
Taxon annotations	349 (201 unique)	224 (125 unique)	514 (248 unique)
Place annotations	219 (115 unique)	419 (154 unique)	401 (139 unique)
Date annotations	64 (63 unique)	60 (59 unique)	92 (90 unique)
Dates in range	July 1905 to April 1907	May 1907 to October 1908	January 1909 to September 1909
Time spent annotating	6 weeks	4 weeks	6 weeks

## Discussion

### Wikisource as a medium for open provisioning and annotation of field notebooks

Our work is part of a larger set of efforts to transcribe, and ultimately mine, the extensive library of historical biodiversity literature (Gwinn and Rinaldo 2009). The choice to use Wikisource for provisioning and annotation of field notes well served our needs, but we recognize the tremendous efforts made by developers to build their own platforms for notebook and journal transcription projects, especially *From The Page* (http://beta.fromthepage.com/), which is being used to transcribe the field notes of renowned herpetologist Lawrence Klauber, of the San Diego Zoo (http://bit.ly/fromthepage-lmk). The primary benefit that *From The Page* offers over Wikisource is that of customization. In the Klauber interface, for instance, developers were able to add a sidebar listing of Klauber’s “slang”: the common names he used to refer to animals in lieu of their scientific names. This could potentially be a great help to volunteer annotators, but is not currently supported by the Wikisource interface.

Wikisource is a relatively new part of the Wikimedia world, and continues to grow to accommodate new uses, as our project demonstrates. The annotation mechanisms we developed were new to Wikisource and pushed the bounds of accepted community practice, especially the relatively obtrusive “link-out boxes” that are placed inline with the text. While there have been some community discussions about the best way of visualizing annotations on Wikisource (e.g., http://bit.ly/N7woun), there has been no major opposition to our templates as yet. We also created community resources to encourage the use of our templates by other notebook annotation projects in the future (see http://en.wikisource.org/wiki/Wikisource:WikiProject_Field_Notes), but, as of this writing, we remain the only field notebook project on Wikisource.

We were able to speedily annotate three notebooks because our crowdsourcing approach worked as well as, or better, than expected, albeit in unexpected ways. Though we attempted to motivate volunteer efforts by promising acknowledgement in this paper and offering a free coffee mug featuring one of Henderson’s field photos in exchange for service, such incentives were ineffective. Instead, two hard-working, anonymous users, known only by IP addresses, completed the majority of annotations. This may indicate that there are motivating factors beyond reward and acknowledgement that spur people to volunteer for these projects.

It is an open question whether using Wikisource fostered or limited participation. There is a learning curve when using Wikimedia products — not just one of learning a new technology, but also of learning the social mores of the existing wiki-community. Potential volunteers and digitization project managers alike may be put off by both barriers to entry, relatively low though they are. On the technology side, we found the Wikisource GUI to be simple and effective, but not always intuitive. For example, despite good help guides, it took some members of our team (who shall remain unnamed) over a month to discover forward and back arrows that allow navigation between sequential notebook pages without returning to the Index. On the social side, posting to the “talk” pages to discuss new policies or initiatives requires learning new ways of communicating with, and integrating into, an online community, which takes time and emotional energy. We wonder if annotator anonymity reflects a desire to avoid entanglement in this community, and simply do a task that is enjoyable.

### Challenges storing and extracting and converting records into interoperable formats

Though Wikisource *can* function as a repository of sorts, it is unclear whether the Wikimedia Foundation *wishes* for it to function as the primary home for digital manifestations of primary source documents. Because there is little easily found documentation describing its long-term digital preservation plans or strategies, we hesitate to call Wikisource a repository. The Wikimedia Foundation may wish to be more deliberate and less opaque in communicating these strategies, especially if it wishes to encourage continued annotation work. Clear digital preservation policies could better assure Wikipedians of their contributions’ relative permanence – whether document uploads, transcriptions or annotations.

We also faced challenges when attempting to capture our workflow in the same structured format as the occurrence records we were extracting: that is, we had more data than we could “fit” into Darwin Core fields. Our solution was to create two sets of files: one composed of simple Darwin Core terms (see supplemental file 2: “dwca-hendersonnotebooks1-3.zip”), and another with a richer set of provenance data showing the process of taxonomic referencing and data processing (see supplemental file 3: “HendersonDwCfull.csv”). This allowed us to present a simple, interoperable dataset while still preserving a record of the densely idiosyncratic process unique to our project and workflow for the purposes of this paper. However, proliferating slightly different versions of this recordset could ultimately cause more confusion than clarity.

Darwin Core’s limited expressivity became especially evident when performing taxonomic referencing; the lack of best practices and vocabularies for describing this multistep process is a notable gap in biodiversity informatics workflows. We particularly note the lack of a VerbatimName term in Darwin Core. Introducing VerbatimName would provide the means to capture the original string as expressed in an occurrence record or field notebook as a starting point to tracking that taxonomic referencing process. Just as VerbatimLocality and GeoreferencingMethod are recorded for future reinterpretation, new terms such as VerbatimIdentification and TaxonResolutionMethod could provide the means to capture essential processing steps as well.

The problems we faced using name resolution services were typical of attempts to automatically extract and parse taxonomic names, thus underscoring the need to better support taxonomic referencing workflows. Though both ITIS and EOL name resolution services returned a substantial number of matches to our names, human validation showed that these resolvers often performed mysteriously, sometimes providing well-resolved binomials when only a genus was entered, or resolving vernacular names in unexpected ways. EOL, for instance, consistently mapped “mouse” to *Amphipyra tragopoginis*, the Mouse Moth. Homonyms across different kingdoms further complicated matters, such as *Crucibulum*, which may be a genus of gastropod or of fungi.

### Challenges with data storage and lasting linkages to sources

Field notebook data and specimen records are often recorded in the field, at the same time, but need to be reconnected after the fact. It is unclear which of Henderson’s observations resulted in collecting events, but re-associating data from these different sources will help enrich local knowledge of biodiversity. A next step will be comparing and contrasting University of Colorado Museum of Natural History zoological specimen catalogs with field notebook observation datasets, both now represented in Darwin Core files. One simple approach is to search on date, and compile taxonomic matches between notebook observations and specimen records. Also of great value will be georeferencing field notebook records to further simplify direct comparisons with other contemporaneous species occurrence records.

We close by noting a final and perhaps most vexing challenge: keeping field note annotations on Wikisource synchronized with the extracted occurrence records. During the occurrence extraction process, we assigned catalog numbers to each occurrence. However, we do not presently have a workflow to then annotate Wikisource with these numbers. Because Wikisource is a necessarily live platform, there is a possibility that additional occurrences will be found and annotated after our initial extraction. Our script, as it is written, would re-catalog these occurrences from the top of the page to the bottom; in short, our catalog numbers are neither stable, nor permanent nor globally unique. This will be hugely problematic if our workflow is implemented in other projects with longer time horizons. In the future, we either need to find a way to annotate occurrences in Wikisource with unique identifiers, or edit our script and cataloging process to remember what we have or have not counted as an occurrence. Although excellent versioning in Wikisource and inclusion of some content from the notebooks in the final CSV files may allow checks for old and new entries, the more stable and reliable solution is to amend the script to automatically annotate references to taxa in Wikisource with such identifiers.

## Appendix 1

Darwin Core categories and field names used in this project. The authors generated the non-Darwin Core Terms and associated fields.

**Table:**

Darwin Core Class	Terms included in Darwin Core file
Record-level Terms	dcterms:modified, basisOfRecord, institutionCode, collectionCode, source
Occurrence	catalogNumber, recordedBy
Event	eventDate, year, month, day, verbatimDate, fieldNotes
Location	country, countryCode, stateProvince, locality, verbatimLocality
Identification	identifiedBy, identificationRemarks,
Taxon	taxonID, scientificName, kingdom, phylum, class, order, family, genus, species, vernacularName, taxonStatus, taxonRemarks
Non-Darwin Core Terms	–ScrapedName records the scientificName for the organism observed as entered by Henderson and transcribed by us.-AnnotatorName records the corrected ScrapedName as recorded by the annotators. The annotators had the option of leaving this field blank, in which case we use the ScrapedName as the AnnotatorName.–Both ScrapedName and AnnotatorName were fed through a taxonomic resolution process (see Methods, section “Proofing the Darwin Core record set”). Three taxonomic resolvers were used for some of the records: the Global Names Index (GNI), the Encyclopedia of Life (EOL) and the Integrated Taxonomic Information System (ITIS). The resulting identifiers and best-matched scientificNames are provided for all three services; additionally, our ITIS service returned vernacular names, which are also recorded. The Source of correct name field indicates whether EOL, ITIS or Both services were returned the correct name.-canonicalScientificName is the scientificName with the authorship information deleted.-AnnotatorLocality: Annotators were asked to provide a corrected, modern place name for the verbatimName; these are recorded here.-Higher taxonomy (kingdom, phylum/division, etc.) were only extracted from ITIS for records where the ITIS name was correct. The taxonID field contains the ITIS Taxonomic Serial Number (TSN) used to look up the higher taxonomy; the scientificName from TSN field contains the scientific name that ITIS associates with that TSN.

## Appendix 2

Data extraction methodology. (doi: 10.3897/zookeys.209.3247.app2) File format: PDF.

**Explanation note:** This supplement contains a detailed description of the steps we carried out to extract transcriptions and annotations, from Wikisource via the MediaWiki API. The Perl scripts we used to carry out these steps are available online at https://github.com/gaurav/henderson.

## Appendix 3

Text file containing all occurrence records. (doi: 10.3897/zookeys.209.3247.app3) File format: CSV.

**Explanation note:** A complete set of occurrence records extracted from Henderson's notebooks 1-3.
